# Gene expression analysis reveals that histone deacetylation sites may serve as partitions of chromatin gene expression domains

**DOI:** 10.1186/1471-2164-6-44

**Published:** 2005-03-23

**Authors:** Liang Chen, Hongyu Zhao

**Affiliations:** 1 Department of Molecular, Cellular and Developmental Biology, Yale University, New Haven, Connecticut, USA; 2 Department of Epidemiology and Public Health, Yale University, New Haven, Connecticut, USA; 3 Department of Genetics, Yale University, New Haven, Connecticut, USA

## Abstract

**Background:**

It has been a long-term puzzle whether chromatin can be further divided into distinct gene expression domains. Because histone deacetylation affects chromatin structure, that in turn may affect the expression of nearby genes, histone deacetylation sites may act to partition chromatin into different gene expression domains. In this article, we explore the relationship between histone deacetylation sites and gene expression patterns on the genome scale using different data sources, including microarray data measuring gene expression levels, microarray data measuring histone deacetylation sites, and information on regulatory targets of transcription factors.

**Results:**

Using 269 *Saccharomyces cerevisiae *microarray datasets, histone deacetylation datasets, and regulatory targets of transcription factors assembled from the Yeast Proteome Database and ChIP-chip data, we found that histone deacetylation sites can reduce the level of co-expression of neighboring genes.

**Conclusion:**

Histone deacetylation sites may serve as possible partition sites for chromatin domains and affect gene expression.

## Background

It is well known that histone acetylation and deacetylation are involved in the transcription process. First, histone modifications can change the affinity of histone proteins to DNA sequences. The disrupted higher order folding of chromatin in turn can affect the transcription process [1,2] because chromosome structural changes can affect accessibility of transcription factors to their target sequences [3,4]. Second, some acetylated lysine sites of histone proteins can function as binding sites for transcription factors [5-7]. Histone deacetylation is also related to aberrant gene expressions in human cancer. For example, in cancer cells, cyclin-dependent kinase inhibitor *p21*^*WAF*1 ^is silenced by the promoter histone hypoacetylation caused by recruited histone deacetylases [8,9]. *p21*^*WAF*1 ^is considered as a *bona fide *tumor-suppressor gene, and its activity can be restored by histone deacetylase inhibitors, which are related to the promoter histone hyperacetylation [9]. Therefore, there is great interest and potential in studying histone acetylation and deacetylation.

The organization of chromatin can be classified into two distinct domains: heterochromatin and euchromatin. The transcription of genes in heterochromatin is usually repressed whereas genes in euchromatin are usually active. Recent studies have shown that the boundaries of heterochromatin are surrounded by high-level histone acetylation [10]. It has been a long-term puzzle whether chromatin can be further divided into distinct gene expression domains, with reports showing that co-expressed genes are clustered into chromatin domains [11-14], which suggests that euchromatin can be further divided into separate domains. The experiments that showed that changing genes' positions may cause aberrant gene regulation also support this hypothesis [15,16]. Recent studies on boundaries and insulators also suggest that chromatin can be partitioned into gene expression sub-domains, because insulators and boundary elements can block the effects of nearby enhancers or nearby heterochromatin [17,18]. Consequently, genes within the blocked domains may be co-regulated through unknown mechanisms. For example, the chicken β-globin HS4 is an insulator in the chromatin region with transitions of histone acetylation levels: from silent chromatin regions with hypoacetylated and Lys-9 methylated histones to active chromatin region with acetylated histones and active genes in erythroid cells [10].

It is well known that there is correlation between histone acetylation level and gene expression [19]. But the detailed relationship between histone acetylation patterns and gene expression domains is less clear. Hypoacetylated histones are associated with silenced chromatin. Histone hyperacetylation may prevent the folding of nucleosomal arrays into more condensed chromatin structures [1,2]. Conversely, histone deacetylation might stabilize higher order chromatin folding. We hypothesize that histone acetylation sites may serve as boundaries of gene expression domains. Although neighboring genes tend to be co-regulated by some enhancers because of their close proximity, higher order chromatin folding caused by histone deacetylation may reduce this neighborhood effect by preventing the distal enhancers from accessing the promoter.

## Results

### Adjacent genes tend to be co-expressed

Although many reports have shown that nearby genes tend to have similar expression profiles [11-14], such observed co-expression may be caused by the spatial arrangement of probes for these genes on microarrays [20]. To minimize this potential spatial effect in the study of co-expression, we used 269 microarray datasets under different conditions because the chip designs differed among different datasets. In order to assess the significance of co-expression between neighboring genes, we performed the hypergeometric test for the pair-wise correlations between adjacent genes and those among all the genes. For every correlation threshold, we counted the number of pair-wise correlations above the threshold for adjacent genes and that for all gene pairs regardless of their physical locations. The number of adjacent genes above a threshold has a hypergeometric distribution under the null hypothesis. The results are summarized in Table 1. As the p-values in this table indicate, the co-expression of neighboring genes is significant for every threshold considered (from 0.5 to 0.9).

**Table 1:** The hypergeometric test for the co-expression effect among neighboring genes. For every correlation threshold, the number of pair-wise correlations for the adjacent genes that are above the threshold was counted. The probability of randomly drawing such high numbers of gene pairs above the threshold from all of the gene pairs is determined by a hypergeometric distribution, as indicated by the p-values.

Total gene pairs	Neighboring gene pairs	Threshold for correlations	Number of total gene pairs above the threshold	Number of neighboring gene pairs above the threshold	P-values based on the hypergeometric tests
18,797,646	6,116	0.5	616,074	752	2.00e-008
		0.6	251,505	401	4.84e-009
		0.7	89,955	196	1.04e-008
		0.8	21,513	68	1.92e-008
		0.9	1,171	13	4.07e-009

### The deacetylation sites reduce correlations among adjacent genes

**Figure 1:**
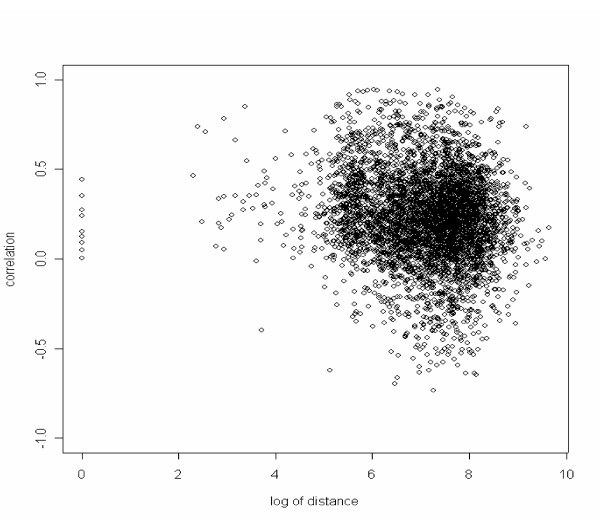
**Scatter plot for the log of physical distances and the expression correlations for neighboring genes without a deacetylation site. **The physical distance is measured by the difference of the start positions (in bp) of the two ORFs. The correlation between the log of physical distances and the expression correlations is -0.140 with the 95% confidence interval (-0.168, -0.111).

**Figure 2:**
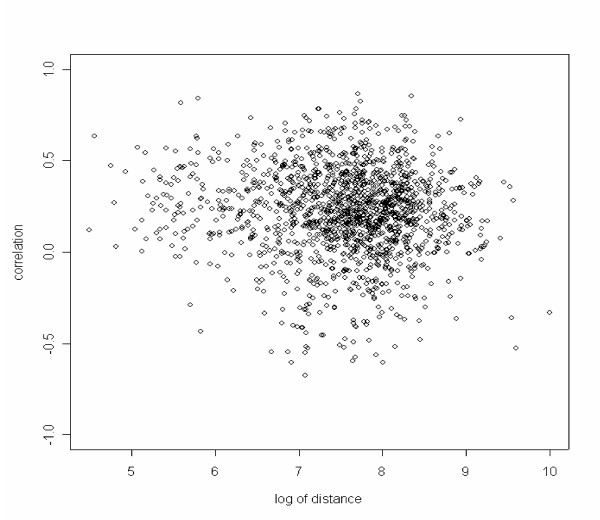
**Scatter plot for the log of physical distances and the expression correlations for neighboring genes with a deacetylation site. **The physical distance is measured by the difference of the start positions (in bp) of the two ORFs. The correlation between the log of physical distances and the expression correlations is -0.074 with the 95% confidence interval (-0.123, -0.024).

In order to test the hypothesis that chromatin deacetylation sites may reduce expression correlations among neighboring genes, we again performed a hypergeometric test. For every correlation threshold, we counted the number of pairwise correlations above the threshold for all adjacent genes and that for all adjacent genes separated by a deacetylation site. The number of adjacent genes with deacetylaiton sites between them above the threshold is hypergeometrically distributed under the null hypothesis. The results in Table 2 suggest that the partition effect is significant at significance level 0.05 for thresholds from 0.5 to 0.9. When a one-sided t-test was performed for correlations between adjacent genes separated by a deacetylation site and those without a deacetylation site, the p-value was 0.0467. This result suggests that the expression correlations between adjacent genes are significantly reduced by the presence of deacetylation sites. In addition, in Figure 1, we plotted the expression correlations versus the physical distances at log scale for the adjacent genes without a deacetylation site. The correlation between the expression similarities and the log of physical distances is -0.140 with the 95% confidence interval (-0.168, -0.111), meanwhile in Figure 2 the correlation for adjacent genes separated by a deacetylation site is -0.074 with the 95% confidence interval (-0.123, -0.024). It indicates that if two neighboring genes are close to each other physically, they are more likely to be co-expressed. However the deacetylation sites can reduce this correlation between the physical distance and the expression similarity.

**Table 2:** The hypergeometric test for the partition effect of the deacetylation sites. For every correlation threshold, the number of pair-wise correlations for the adjacent genes separated by deacetylation sites that are above the threshold was counted. The probability of randomly drawing such low numbers of gene pairs above the threshold from all of the adjacent gene pairs is determined by a hypergeometric distribution.

Total neighboring gene pairs	Neighboring gene pairs separated by deacetylation sites	Threshold for correlations	Number of neighboring gene pairs above the threshold	Number of neighboring gene pairs separated by deacetylation sites above the threshold	P-values based on the hypergeometric tests
6,116	1,558	0.5	752	161	0.00322
		0.6	401	73	0.000231
		0.7	196	26	0.0000167
		0.8	68	5	0.000120
		0.9	13	0	0.0218

### Tests on assembled co-regulation groups from YPD and ChIP-chip experiments

Because both transcription factors and chromatin status can affect gene expression levels, we further studied the effects of chromatin status on genes sharing common regulators. We assembled 294 co-regulation groups from YPD (Yeast Proteome Database as of March 2004 [21]) and calculated the pair-wise correlations within each group. The co-regulation group was defined by the target genes of a regulator. The deacetylation sites through the genome were used to partition the genes into different domains. There are 24,833 gene pairs sharing one common regulator. Among them, 141 pairs of genes are in the same deacetylation partition group, and 46 pairs of genes belong to neighboring deacetylation partition groups. We performed a hypergeometric test for the 141-pair group versus all of the pair-wise genes, and the p-values were significant (Table 3). We also performed a hypergeometirc test for the 46-pair group and all of the pair-wise genes and the p-values were not significant compared with the 141-pair group (Table 3). The results show that genes sharing common regulators are more co-expressed if they are in the same deacetylation partition groups. We also performed a one-sided t-test for correlations between the genes sharing regulators and deacetylation partition groups and those sharing regulators but in neighboring deacetylation partition groups, and the p-value was 3 × 10^-4^. This suggests that there is significant evidence that the correlations between genes sharing regulators and deacetylation partition groups are greater than those between genes sharing regulators but in neighboring groups. In other words, in addition to sharing common regulators, belonging to the same deacetylation partition group may be another factor contributing to the co-expression of two genes.

**Table 3:** The hypergeometric tests for the partition effect of the deacetylation sites for the co-regulated (YPD) gene pairs. For every correlation threshold, the number of pair-wise correlations for the genes with a common regulator and in the same partition group that are above the threshold was counted. The number of pair-wise correlations for the genes with same regulator and in the neighboring partition groups that are above the threshold was also counted. The probability of randomly drawing such high numbers of gene pairs above the threshold from all of the gene pairs with same regulator is determined by a hypergeometric distribution. The p-values are indicated in the table.

Total gene pairs sharing one regulator	Gene pairs sharing one regulator and both are in the same deacetylation partition group	Threshold for correlations	Number of gene pairs sharing one regulator above the threshold	Number of gene pairs sharing one regulator and both are in the same deacetylation partition group above the threshold	P-values based on the hypergeometric tests
24,833	141	0.5	4,676	80	3.43e-011
		0.6	2,925	54	2.37e-011
		0.7	1,519	45	2.24e-011
		0.8	422	20	2.92e-011
		0.9	30	3	0.0000243

Total gene pairs sharing one regulator	Gene pairs sharing one regulator and they belong to neighboring deacetylation partition groups	Threshold for correlations	Number of gene pairs sharing one regulator above the threshold	Number of gene pairs sharing one regulator and they belong to neighboring deacetylation partition groups above the threshold	P-values based on the hypergeometric tests

24,833	46	0.5	4,676	12	0.0784
		0.6	2,925	8	0.0853
		0.7	1,519	8	0.00165
		0.8	422	2	0.0432
		0.9	30	0	0.0541

In addition to YPD data, we also used ChIP-chip data [22] to define regulatory targets of transcription factors. A p-value threshold of 0.005 was used to infer the binding of regulators for each gene. There are 476,649 gene pairs sharing at least one regulator based on this threshold, and we calculated the pair-wise correlations for these pairs. Among all the pairs, 1,811 pairs belong to the same deacetylation partition group, and 1,019 pairs are in the neighboring partition groups. The comparisons among different groups are summarized in Table 4. For the genes in the same deacetylation partition group, the proportion of the number of pairs having correlations above each threshold is significantly higher than that based on all gene pairs. But for the genes in the neighboring deacetylation partition groups, the difference is not statistically significant.

**Table 4:** The hypergeometric test for the partition effect of the deacetylation sites for the co-regulated (ChIP-chip experiments) gene pairs. For every correlation threshold, the number of pair-wise correlations for the genes with the same regulator and being in the same partition group that are above the threshold was counted. The number of pair-wise correlations for the genes with the same regulator and being in the neighboring partition groups that are above the threshold was also counted. The probability of randomly drawing such numbers of gene pairs above the threshold from all of the gene pairs with the same regulator is determined by a hypergeometric distribution.

Total gene pairs sharing one regulator	Gene pairs sharing one regulator and both are in the same deacetylation partition group	Threshold for correlations	Number of gene pairs sharing one regulator above the threshold	Number of gene pairs sharing one regulator and both are in the same deacetylation partition group above the threshold	P-values based on the hypergeometric tests
476,649	1,811	0.6	14,738	134	<4.75e-011
		0.7	8,419	68	5.71e-009
		0.8	4,556	29	0.00329
		0.9	621	7	0.00293

Total gene pairs sharing one regulator	Gene pairs sharing one regulator and they belong to neighboring deacetylation partition groups	Threshold for correlations	Number of gene pairs sharing one regulator above the threshold	Number of gene pairs sharing one regulator and they belong to neighboring deacetylation partition groups above the threshold	P-values based on the hypergeometric tests

476,649	1,019	0.6	14,738	34	0.287
		0.7	8,419	18	0.438
		0.8	4,556	8	0.638
		0.9	621	1	0.383

**Figure 3:**
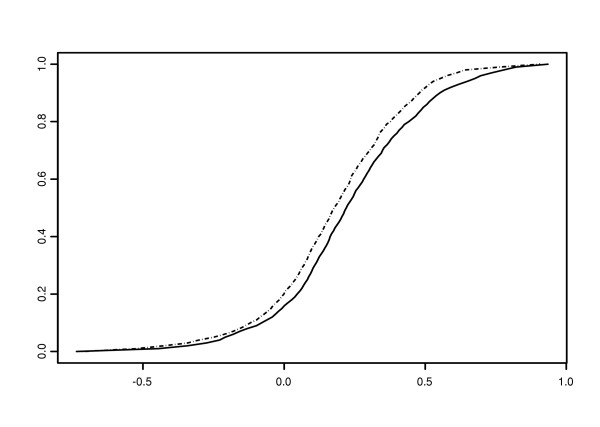
**Comparison of the empirical cumulative distributions of the two samples. **The x-axis indicates the pair-wise correlation. The y-axis indicates the empirical cumulative distribution of the pair-wise correlation. The solid line is for the correlations between genes sharing one regulator and being in the same deacetylation partition group (the 1,811-pair sample). The dotted line is for the correlations between genes sharing one regulator and belonging to neighboring deacetylation partition groups (the 1,019-pair sample).

When we performed a one-sided t-test for the correlations for the 1,811-pair sample and the 1,019-pairs sample, the p-value was 4 × 10^-8^. In Figure 3, we compared the empirical cumulative distributions of the two samples. It can be seen that the distributions of the correlations are different, and there are more pairs having higher pair-wise correlations for the 1,811 pair group belonging to the same deacetylation partition than that for the group belonging to the neighboring partitions. A one-sided two-sample Kolmogorov-Smirnov test was performed between these two samples, and there is statistically significant evidence suggesting that the two distributions are different (p-value < 0.0001). The Kolmogorov-Smirnov test is a test of whether the two data samples come from the same distribution, without making assumption about the distribution of the data.

### Study on gene expression domains

**Figure 4:**
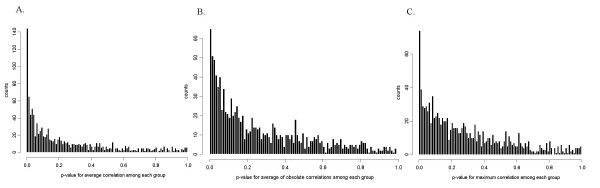
**The histograms of the p-values for the correlations in partition groups. **(A) The histogram of the p-values for the average of pair-wise correlations in partition groups. (B) The histogram of the p-values for the average of absolute pair-wise correlations in partition groups. (C) The histogram of the p-values for the maximum pair-wise correlations in partition groups. The p-values are based on randomly sampling from the whole genome 1,000,000 times for each partition group size.

In addition to examine neighboring genes, we also studied all genes in the same partition group simultaneously. For each partition group, we calculated the average correlation, the average of absolute correlations, and the maximum correlation. To test the significance of the correlations, we sampled groups of genes from the genome for each group size at random 1,000,000 times. For each sampled group, the average correlation, the average of absolute correlations, and the maximum correlation were calculated. The p-values can be estimated based on the correlations for these simulated groups. The results summarized in Figure 4 clearly demonstrate the statistical significance for the high correlations for the genes grouped by deacetylation sites.

Because the presence of such clusters may be due to reasons other than histone deacetylated sites, e.g. spatial proximity among neighbouring genes, we also performed a different type of simulations to assess the significance of the maximum correlation within each group. We permuted the order of the groups on the whole genome 900,000 times. For each permuted sample, the number of groups that had the maximum correlation larger than 0.6, 0.7, 0.8, or 0.9 was counted. We called these groups with the maximum correlation above a given threshold as qualified groups. We counted the number of permuted samples with more qualified groups than that based on the observed histone deacetylation partition. The statistical significance of the observed partition is estimated as the proportion of the permuted samples having more qualified groups than that based on the observed histone deacetylation partition. As shown in Table 5, the results are statistically significant for the threshold of 0.6, 0.7, and 0.8. But the results for the average correlation and the average of absolute correlations were not statistically significant (data not shown). One possible reason is that many other regulators can affect the transcription process within each partition, resulting in less significant results.

**Table 5:** The significance of the number of qualified groups for histone deacetylation partition. The statistical significance of the histone deacetylation partition is estimated by the proportion of permutations that have more groups with the maximum correlation larger than the threshold than the observed histone deacetylation partition.

Threshold	Observed qualified groups for maximum correlations	Number of times of having more qualified clusters for simulations	P-values
0.6	352	27,795	0.0309
0.7	198	26,285	0.0292
0.8	77	6,536	0.00726
0.9	17	61,006	0.0678

In order to test whether there is function enrichment for the deacetylation partition groups, the following procedure was performed. There are 1,113 deacetylation partition groups with more than one gene in total. For each of these deacetylation partition groups, the Yeast GO Slim terms were mapped to the genes using SGD Gene Ontology Slim Mapper [23]. The p-value was calculated using the hypergeometric distribution as the probability of n or more out of m genes having a specific GO Slim term, given that N out of M genes have that GO slim term in the whole genome. If there was at least one GO Slim term with p-value less than or equal to 0.0001 in a deacetylation partition group, we called it a significant group. There are 386 significant groups out of the 1,113 deacetylation partition groups, whereas only 4 out of 100 simulations have more than 386 significant groups. The simulation was performed by the group order permutation as described in the above paragraph. If we pool all of the p-values from the simulations together, the 5% percentile rank is 3.02 × 10^-4 ^and 493 out of 1,113 groups have at least one GO Slim term with p-value less than or equal to 3.02 × 10^-4^. These results indicate that the deacetylation partition groups are enriched for genes of specific GO Slim terms.

## Discussion

The effect of chromatin status on gene transcription regulation is well known, but the detailed mechanisms remain elusive. In this paper, we tested the hypothesis that histone deacetylation takes part in the chromatin expression domain formation through combined analyses of different data types, including microarray data for deacetylation sites, gene expression, ChIP-chip data, and YPD database. These analyses have revealed significant effects of histone deacetylation on gene expression patterns. Although we only presented results using 1.95 as the threshold for defining the deacetylation sites (which was used in the ref [24]), different thresholds (e.g. 1.8 and 2.1) yield similar results.

As an example for the effect of deacetylation sites, we considered two genes, YLR329W and YLR328W, which are separated by a deacetylation site in our analysis. YLR329W (REC102) is a protein involved in early stages of meiotic recombination. The early meiosis-specific genes are repressed by histone deacetylation in the mitotic cell cycle. They are activated by histone acetylation in the meiotic stage [25]. The correlation between these two genes expression profiles is 0.1965. The correlation increases to 0.7499 in the sporulation time course dataset [26] which consists of meiosis. This example illustrates that these two genes are co-expressed when there is no deacetylation between them, while the correlation is decreased by the presence of a deacetylation site.

The observed co-expression among neighboring genes in the yeast cell cycle datasets are still debatable because the co-expression may be due to spatial arrangement of the probes on microarrays [20]. In our study, we used 269 microarray datasets under different conditions. Because the chip designs differed among different datasets, the spatial effect is significantly minimized when combining a large number of datasets of diverse sources. More importantly, the results that compare the genes in the same deacetylation partition and those in neighboring partitions demonstrate that the spatial effect is not the reason for the observed difference. This is because the spatial effect should have similar impact under both conditions. This is further supported by the permutation results of the partition groups.

In addition to histone acetylation and deacetylation, DNA methylation is another key factor for the epigenetic effects on gene expression. Because *Saccharomyces cerevisiae *has no detectable DNA methylation, this organism serves as a good model to examine the relationship between deacetylation patterns and gene expression domains.

Since histone deacetylation was measured in a heterogeneous population, the domain partitions may not be an accurate representation for a specific condition. In addition, other histone modifications that can affect gene expressions may be ignored and need further studies.

## Conclusion

The fact that histone modification may affect gene transcription has been recognized for many years. Thanks to the high-throughput gene expression microarray data and gene acetylation microarray data, we are able to study the effects of histone acetylation at the whole genome scale. In this article, we confirmed the previous findings on the co-expression effect of neighboring genes. Furthermore, we have tested the hypothesis that histone deacetylation sites serve as possible partitions for chromatin domains of gene expression through several approaches. We hypothesize that histone deacetylation can lead to dense higher order chromatin structure and reduce the accessibility of the transcription factors to their target sequences. The detailed mechanisms need to be further studied through biological experiments.

## Methods

### Histone deacetylation sites and gene expression data

In order to test our hypothesis that deacetylation sites function as a boundary complex and partition the neighborhood gene expression domains, we correlated histone deacetylated sites with gene expression profiles as follows. The histone deacetylation sites were inferred through specific histone acetylation microarrays [24], where chromatin immunoprecipitation and DNA microarrays are combined to determine the histone acetylation levels. The increased histone acetylation sites in the absence of histone deactylases were considered as the putative histone deacetylation sites (1.95 fold change as cutoff) in the wild type strain. The data for the histone deacetylases RPD3, HDA1, HOS1, HOS2, HOS3 and SIR2 were combined for the intergenic regions. These data are normalized average acetylation fold changes. The intergenic region was considered as a putative deacetylation site if one dataset for any of the histone deacetylases is larger than 1.95. In total, there are 1,747 putative deacetylation sites. The putative sites throughout the genome were used to define gene cluster domains. We used 269 yeast microarray datasets (downloaded from NCBI GEO database [27], there are 6,132 genes with <=25% missing data across all of the experiments) for gene expression analysis. In total, there are 1574 deacetylation groups. Among them, 461 deacetylation groups only contain one gene, and 315, 183, 149 and 121 groups contain 2, 3, 4, and 5 genes. The detailed group information is summarized in Additional File 1.

### Statistical analyses

Pearson's correlation coefficients were calculated for gene expression data. The hypergeometric tests were performed to assess the significance of the co-regulation effects between neighboring genes, the significance of the partition effect of the chromatin deacetylation sites, and the effects of chromatin status on genes sharing common regulators. One-sided t-tests were performed to compare the genes in the same deacetylation partition groups and those in neighboring partition groups. The one-sided two-sample Kolmogorov-Smirnov test was performed to test the difference between the distribution of correlations for genes sharing the same regulators and deacetylation partition groups and that for genes sharing the same regulators but are in neighboring groups.

## Authors' contributions

LC carried out the data analyses and was involved in the preparation of the manuscript. HZ supervised the study and was involved in the preparation of the manuscript. Both authors read and approved the final manuscript.

## Supplementary Material

Additional File 1 **Deacetylation partition groups information. **Groups.els is an excel spreadsheet which includes the gene names and the chromosome number for each deacetylation partition group. Click here for file
